# Bees and flowers: How to feed an invasive beetle species

**DOI:** 10.1002/ece3.5217

**Published:** 2019-05-14

**Authors:** Jérémy Gonthier, Anna Papach, Lars Straub, Joshua W. Campbell, Geoffrey R. Williams, Peter Neumann

**Affiliations:** ^1^ Vetsuisse Faculty, Institute of Bee Health University of Bern Bern Switzerland; ^2^ Swiss Bee Research Centre Agroscope Bern Switzerland; ^3^ Department of Entomology & Plant Pathology Auburn University Auburn Alabama

**Keywords:** *Aethina tumida*, feeding preferences, host shift, solitary bees

## Abstract

Invasive species may exploit a wide range of food sources, thereby fostering their success and hampering mitigation, but the actual degree of opportunism is often unknown. The small hive beetle (SHB), *Aethina tumida*, is a parasite of honeybee colonies endemic to sub‐Saharan Africa. SHBs have now spread on all habitable continents and can also infest colonies of other social bees. To date, the possible role of solitary bee nests as alternative hosts is unknown. Similarly, flowers as possible alternative food sources are not well understood. Here, we show that SHBs can complete an entire life cycle in association with nests of solitary bees *Megachile rotundata*. The data also show that flowers can serve as alternative food sources. These results support the opportunistic nature of this invasive species, thereby generating further obstacles for mitigation efforts in the field. It also suggests that SHB invasions may result in more serious consequences for endemic bee fauna than previously thought. This provides further motivation to slow down the global spread of this pest, and to improve its management in areas, where it is established.

## 1 INTRODUCTION

Invasive species are becoming more and more frequent due to globalization and increased inter‐regional transport of goods (Cassey, Blackburn, Duncan, & Chown, 2005; Mooney & Cleland, 2001). In many cases, their introduction to an area can cause dramatic changes to community and ecosystem structure (Gurevitch & Padilla, 2004). For example, introduced species can parasitize new native hosts which may not have evolved defenses against the invader due to lack of co‐evolutionary history. Honeybees and other insect pollinators were not spared by this phenomenon, as they too can be strongly affected by new parasites (Neumann, Pettis, & Schäfer, 2016; Rosenkranz, Aumeier, & Ziegelmann, 2010).

The small hive beetle (SHB), *Aethina tumida* Murray (Coleoptera: Nitidulidae) is an example of such an invasive species (Neumann & Elzen, 2004). It is a long‐known parasite and scavenger of honeybee colonies endemic to sub‐Saharan Africa (Lundie, 1940) and has now spread to all habitable continents (Al Toufailia et al., 2017; Granato et al., 2017; Lee et al., 2017; Muli, Kilonzo, & Sookar, 2018; Neumann et al., 2016). Within honeybee colonies, SHBs feed on honey, pollen as well as bee brood and adults and/or are fed trophallactically by the bees (reviewed by Neumann et al., 2016). The adult SHBs mate and oviposit in the bee nests; the emerging larvae feed on all available food stuff, which often results in the full structural collapse of the entire nest, and then leave the colonies for pupation in nearby soil (reviewed by Neumann et al., 2016). Adults emerging from the soil infest new host colonies, thereby completing the life cycle of *A. tumida* (Neumann et al., 2016). In its endemic range, SHB is usually considered a minor pest of honeybee colonies (Neumann & Elzen, 2004). However, in the new invasion ranges, SHB can considerably impact local honeybee populations (Hood, 2004; Neumann & Elzen, 2004). Evidence so far suggests that SHB is opportunistic, and able to take advantage of various alternative food sources and host species (Buchholz et al., 2008; Neumann et al., 2016).

In the past decade, several new observations of other social bees acting as alternative hosts have been reported (bumblebees: *Bombus impatiens*: Spiewok & Neumann, 2006; Hoffmann, Pettis, & Neumann, 2008; stingless bees: *Austroplebeia australis*, Halcroft, Spooner‐Hart, & Neumann, 2011; *Dactylurina staudingeri,* Mutsaers, 2006; *Melipona beecheii*, Lóriga Peña, Fonte Carballo, & Demedio Lorenzo, 2014; *Tetragonula carbonaria*, Greco et al., 2010; Wade, 2012). Since nests of solitary bees also contain nectar, pollen, and bee brood similar to social bee colonies (Michener, 2000), it may well be that they can serve as alternative hosts of SHBs too. Therefore, the possibility of infestation of solitary bee nests by SHBs was investigated for the first time in semifield and field experiments. The purpose of this work is to help evaluate the potential threat of SHB to solitary bees and whether they may act as reservoirs for this pest of social bees.

We chose the leafcutter bee *Megachile rotundata* as a model organism for solitary bees mainly because it is commercially available due to being an efficient pollinator (Michener, 2000), for example, for alfalfa pollination (“alfalfa leafcutter bee”). It is native to Europe, but has been introduced to all continents except for Antarctica (Michener, 2000), thereby naturally overlapping with the new distribution ranges of *A. tumida* (Neumann et al., 2016). This bee species is partially bivoltine (Pitts‐Singer & Cane, 2011). Each mated *M. rotundata* female takes advantage of available cavities for constructing her nest using cut plant leaves, which consists of 20–50 cells with ~90–94 mg of diet each (nectar/pollen in a 2:1 ratio, Pitts‐Singer & Cane, 2011). Adult offspring emerge and mate, thereby completing the life cycle of *M. rotundata* (Pitts‐Singer & Cane, 2011). Assuming that SHBs can actually take advantage of *M. rotundata* nests to complete a whole life cycle, a few points should be considered. Individual SHBs need ~25 mg of diet (honey/pollen paste 1:2 ratio, Neumann et al., 2013) from the egg to the postfeeding wandering larvae stage (Neumann, Peter & Pettis, Jeff, unpublished data). Due to the different proportions of carbohydrates versus proteins in the solitary bee and beetle diets, the limiting factor for SHB reproduction in association with *M. rotundata* would most likely be the available pollen in the bee nests (29.7–33.84 mg/cell, Pitts‐Singer & Cane, 2011). Since one female leafcutter bee produces between 20–50 eggs per season (Pitts‐Singer & Cane, 2011) and one SHB needs 16.67 mg of pollen (Neumann, Peter & Pettis, Jeff, unpublished data), one *M. rotundata* nest can in theory provide sufficient food for 36–101 SHBs to reach the postfeeding stage, when excluding possible preying on the bee larvae (e.g., SHB larvae are known to prey on honeybee larvae, Neumann et al., 2016). Given that adult SHBs infest *M. rotundata* nests, a comparison between these predicted values (1–2 adult SHB offspring per infested *M. rotundata* cell) and the actual observed reproduction will provide a valuable proxy for the nature of this species interface.

In the second part of this study, we evaluated the role of flowers as an alternative food source for SHBs. Several studies have shown that SHBs can use fruits and even rotten meat as alternative food sources (Buchholz et al., 2008; Ellis, Neumann, Hepburn, & Elzen, 2002; Neumann & Elzen, 2004). Since flowers produce nectar and pollen, one base of SHB food regime, they may be used as alternative food sources too. So far, evidence suggests that SHBs are unlikely to visit flowers (Buchholz et al., 2008; Willcox, Howlett, & Neumann, 2017). However, the diversity of plants tested remains scarce, thus calling for further investigation to clarify the use of flowers as an alternative food for SHBs. We here determined the rate of foraging and survival of SHBs on blooming plants and conducted an experiment to investigate if SHBs forage on flowers in semifield conditions. By evaluating the possible role of both solitary bees and flowers, we increase our knowledge about the level of opportunism of this invasive species, which is essential for adequate SHB mitigation (Schäfer et al., 2019).

## 2 METHODS

The study was conducted from April‐September 2018 at the Bee Laboratory, Auburn University, USA. Experimental SHBs were obtained from a laboratory rearing program that was established using field‐sampled adults and standard protocols (Neumann et al., 2013). The solitary communal nesting bee *Megachile rotundata* and associated nesting material were obtained from Crown Bees (www.crownbees.com).

### 2.1 Infestation experiments

#### 2.1.1 Experiment 1. Can *Aethina tumida* oviposit and complete its lifecycle in association with *Megachile rotundata* nests?

**Figure 1:**
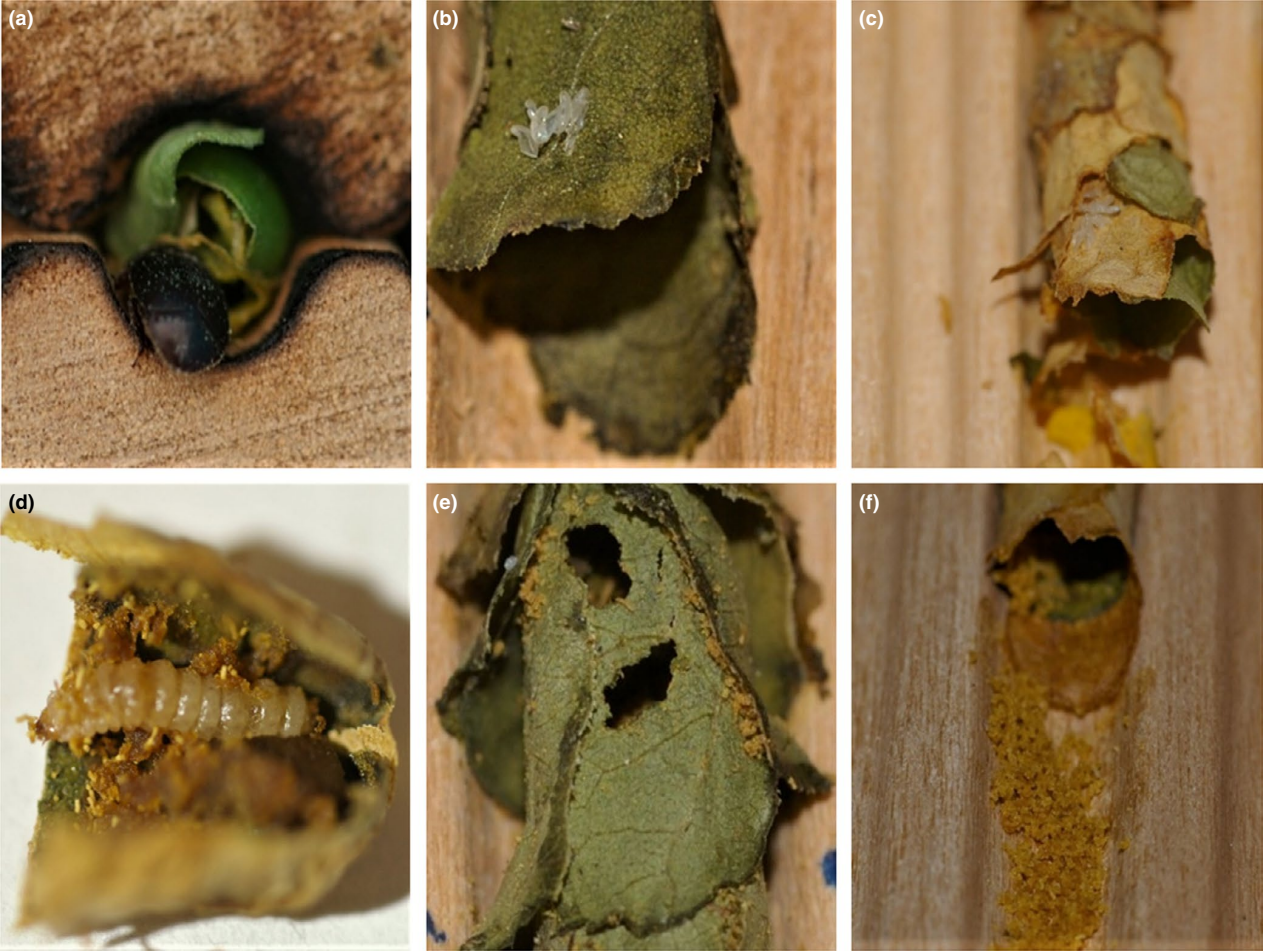
Infestation of *Megachile rotundata* nests by small hive beetles: (a) adult entering the nest; (b) egg clutch on a cell; (c) empty eggshells on a cell; (d) larvae foraging in a cell; typical examples of clinical symptoms: (e) parts of leaf eaten; (f) cell open and pieces of pollen distributed in front

Preliminary artificial infestations were required to test if SHBs can in principle infest *M. rotundata* nests. Given that solitary bee nests can serve as a breeding ground, adult SHBs should oviposit eggs in nests that will eventually complete their lifecycle. To investigate oviposition, at dusk (the natural time window for SHB flight, Neumann & Elzen, 2004) 20 *M. rotundata* nest tubes that contained between three to 10 cells (cell partitions containing bee larvae/pupae) received 20 mated SHB females at each of their entrances. The beetles were confined to the entrances of the nest tubes for 2 hr to settle down by taping the entrance. Oviposition and clinical symptoms of infestation were assessed by dissecting the nests 16 days later, the maximum time for SHB eggs to hatch and larvae to develop under the given local environmental conditions (Neumann et al., 2016). The nests were carefully examined for clinical symptoms of SHB infestation (Figure 1): (a) Presence of SHB eggs or empty egg shells; (b) Presence of SHB larvae; (c) Part of the cell eaten; (d) Cell open; and (e) Pollen randomly distributed.

Additionally, development of SHB larvae was investigated by introducing 10–20 freshly laid eggs (<24 hr old) at the front of 20 solitary bee nest tubes that each contained between three to 10 cells. For this test, we used observatory nests from the company Mauerbienenzucht (www.mauerbienen.com). Observations of larvae or any clinical symptoms of infestation (Figure 1) were conducted over a 5 day period, because all SHB eggs should have emerged by then (Neumann & Elzen, 2004).

Next, to investigate successful pupation, a plastic box (40 × 40 × 30 cm) containing adequate soil for SHB pupation (Neumann et al., 2013) was placed below the nests used to test oviposition in order to collect any SHB wandering larvae emerging from above. After 16 days, the pupation box was placed in an incubator at +25°C and 80% RH in constant darkness (Neumann et al., 2013) and checked every 2 days for 45 days for adult emergence.

#### 2.1.2 Experiment 2. Will *Aethina tumida* infest and complete its lifecycle in association with *Megachile rotundata* nests under semifield conditions?

**Figure 2:**
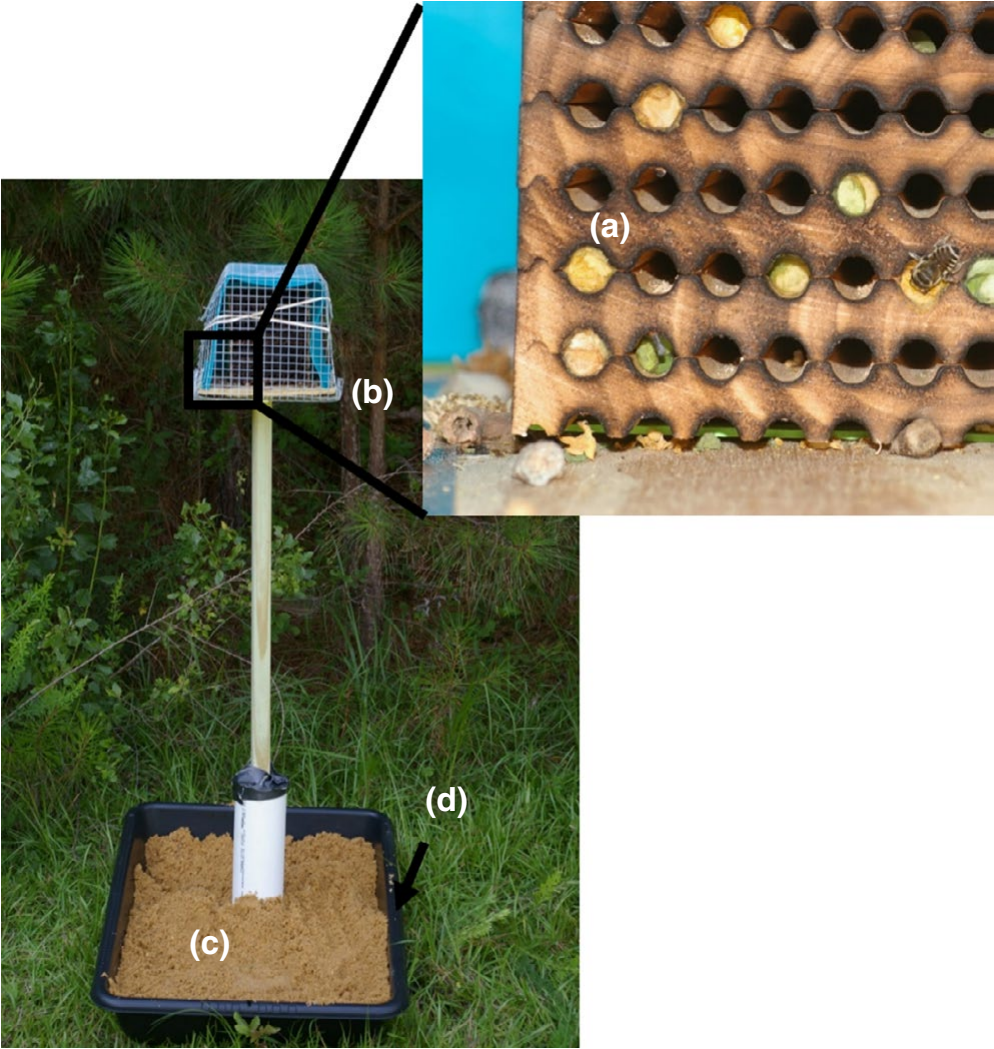
Set‐up of semifield experiment 2 and field experiment 3: (a) Woodblock with holes (∅ = 6 mm) with an adult *Megachile rotundata* and nine nests.; (b) covered platform (30 × 30 cm) 1.2 m above ground; (c) pupation box (40 × 60 × 25 cm) with suitable soil for small hive beetle pupation; and (d) insect glue circle on the outside of the box to limit ant predation and prevent escape of SHB larvae

Experiments were conducted during June and July 2018. A total of 80 *M. rotundata* (40 females, 40 males) were released into each of six tents (Magellan Outdoors^®^ 4.26 × 3.65 m Deluxe Screen House), which were each equipped with flowering buckwheat, *Fagopyrum esculentum* (15 pots, ∅ = 40 cm) and nesting material (Figure 2). The nesting material was composed of a wooden block with 78 holes (∅ = 6 mm) in a covered platform (30 × 30 cm). The platform was fixed 1.2 m above a pupation box (40 × 60 × 25 cm) containing loose and sandy soil for SHB pupation (Neumann et al., 2013). The pupation box was circled with insect glue (Tree Tanglefoot^®^ Insect Barrier Tub) on the outside to ward off possible ant predation and to prevent escape of SHB wandering larvae. The bees were left alone for 2 weeks to provide them with the opportunity to emerge from their cocoons and establish nests. At day 15, SHB infestation was investigated by introducing at dusk 50 adult SHBs from the laboratory rearing (sex ratio female to male 2:1) into five of the six tents. One tent was kept as a negative control. Every 2 days, nests were visually inspected over 5 min each for the presence of any SHB life stages and/or clinical symptoms of infestation (Figure 1; Neumann & Elzen, 2004). After 36 days, the nests were collected at dusk, frozen at −20°C and dissected to assess any clinical symptoms of SHB infestation. A nest was considered a tube with at least one cell. Additionally, pupation boxes containing soil were collected, covered with insect mesh and checked for adult SHB emergence over 45 days. Then, the sand was sieved to find any possible remaining SHBs that had not yet emerged from the soil (Mürrle & Neumann, 2004).

#### 2.1.3 Experiment 3. Will *Aethina tumida* infest and complete its lifecycle in association with *Megachile rotundata* nests under field conditions?

Experiments were conducted at five locations in managed SHB‐infested local apiaries (<3 m from the hives). One additional location served as a negative control and was >3 km away from any apiary. A total of 250 cocoons of *M. rotundata* (sex ratio female to male 1:1.2; Pitts‐Singer & James, 2005) ready to emerge were released at each of the six locations equipped with the same nest structure as for the semifield experiment; however, the wooden block contained more holes (157 (∅ = 6 mm); Figure 2). The nests were visually inspected for the presence of SHBs every 4 days. After 36 days, the nests were collected at dusk, frozen and dissected to inspect for clinical symptoms of SHB infestation (Figure 1; Neumann & Elzen, 2004). The pupation boxes containing soil were collected, covered with insect mesh, and checked for adult SHB emergence for 45 days. Then, the sand was sieved to find any possible remaining SHBs that had not yet emerged from the soil (Mürrle & Neumann, 2004).

### 2.2 Association with flowers

#### 2.2.1 Experiment 4. Can flowering plants serve as an alternative food source for *Aethina tumida*?

Five different plant species were tested as a potential alternative food source of SHBs. We selected plants growing locally in Auburn, Alabama that were flowering during the experimental time. These plants are also known to be forage for various local beetle species (Joshua W Campbell, personal observations). In the laboratory, the following trials (*N* = 3 replicates each) were arranged in cylindrical plastic containers (∅ = 15 cm; *h* = 30 cm): (a) *Fagopyrum esculentum* "buckwheat"; (b) *Lagerstroemia indica* "crepe myrtle"; (c) *Magnolia grandiflora* "sweetbay magnolia"; (d) *Coreopsis tinctoria* "Golden Tickseed"; (e) *Erigeron annuus* "annual fleabane"; (f) pollen and honey paste (30 g; 1:1) (=positive control); and (g) no food (=negative control). Each container was closed with an insect mesh (∅ = 1.0 mm). The flowering plants were placed inside a water bottle. To investigate SHB oviposition, standard oviposition sites were used (Neumann et al., 2013). SHB adults (*N* = 10; >10 days old, maintained with honey water) were transferred from the laboratory rearing (sex ratio female to male 2:1) to each container. All containers were stored in the laboratory at a constant 24°C and normal daylight (Neumann et al., 2013). Flowers were sprayed with water on a daily basis and to ensure they remained fresh; they were replaced after 3 days. Adult SHB mortality, foraging intensity (% of live beetles observed on flowers) and oviposition were assessed daily; dead beetles removed to limit cannibalism (Neumann et al., 2016). The experiment was terminated after 19 days, when all individuals of the negative control had died.

#### 2.2.2 Experiment 5. Will *Aethina tumida* forage on buckwheat flowers, *Fagopyrum esculentum*, under semifield conditions?

While the infestation experiment 2 in the tents took place, visual observations were conducted every 2 days in the afternoon during 10 min in each tent to quantify any SHBs on the buckwheat flowers. The observations started 10 days after releasing the adult SHBs and lasted 20 days.

### 2.3 Statistical analyses

All statistical tests and figures were performed using NCSS 12 (NCSS, 2018). Data were tested for normality using the Shapiro–Wilk's test, and visual inspections of the data were made using Q‐Q‐plots. We applied a logistic regression model to test for significant difference for numerous binary response variables in experiment 1.1. The treatment (control or SHB infested) was included as the fixed factor, where as individual tents and nests were included as random effects (Peng, Lee, & Ingersoll, 2002). An XY scatter plot and Spearman's correlation coefficient were used to assess possible relationships among the number of cells produced and the number of cells with clinical SHB symptoms. Survival analyses on flowers were conducted by using Kaplan–Meier survival curves and Logrank tests (Mantel–Haenszel test) with multiple pairwise corrections (Bonferroni test [Bmtc]). Mean cumulative survival differences and associated standard deviations were calculated 19 days after start of experiment. Furthermore, when normality was rejected (Shapiro–Wilk's test, *p* < 0.05), as in the case for foraging intensity between flowers (calculated daily per cage by dividing the number of beetles on the flowers by the total number of living beetles), data were compared by using a nonparametric Kruskal–Wallis multiple comparison One‐Way ANOVA with an additional post hoc test (Bmtc). Median foraging intensity, as well as their 95% confidence intervals (CI), was then calculated. Lastly, a XY scatter plots and Spearman's correlation coefficients were used to assess possible relationships between survival and foraging intensity of adult SHBs on flowers.

## 3 RESULTS

### 3.1 Infestation experiments

#### 3.1.1 Experiment 1. Can *Aethina tumida* oviposit and complete its lifecycle in association with *Megachile rotundata* nests?

Sixteen days after artificial infestation with adult SHBs, nine of the 20 nests exhibited clinical SHB symptoms (Figure 1). Of the nine nests showing clinical SHB symptoms, two nests revealed empty SHB eggshells (Figure 1).

Five days after artificial infestation with SHB eggs, six of the 20 nests showed clinical SHB symptoms (Figure 1). Of the six, three had SHB larvae of different developmental stages including those close to the wandering stage (Neumann & Elzen, 2004). Lastly, a total of four adult SHBs emerged after soil incubation.

#### 3.1.2 Experiment 2. Will *Aethina tumida* infest and complete its lifecycle in association with *Megachile rotundata* nests under semifield conditions?

**Figure 3:**
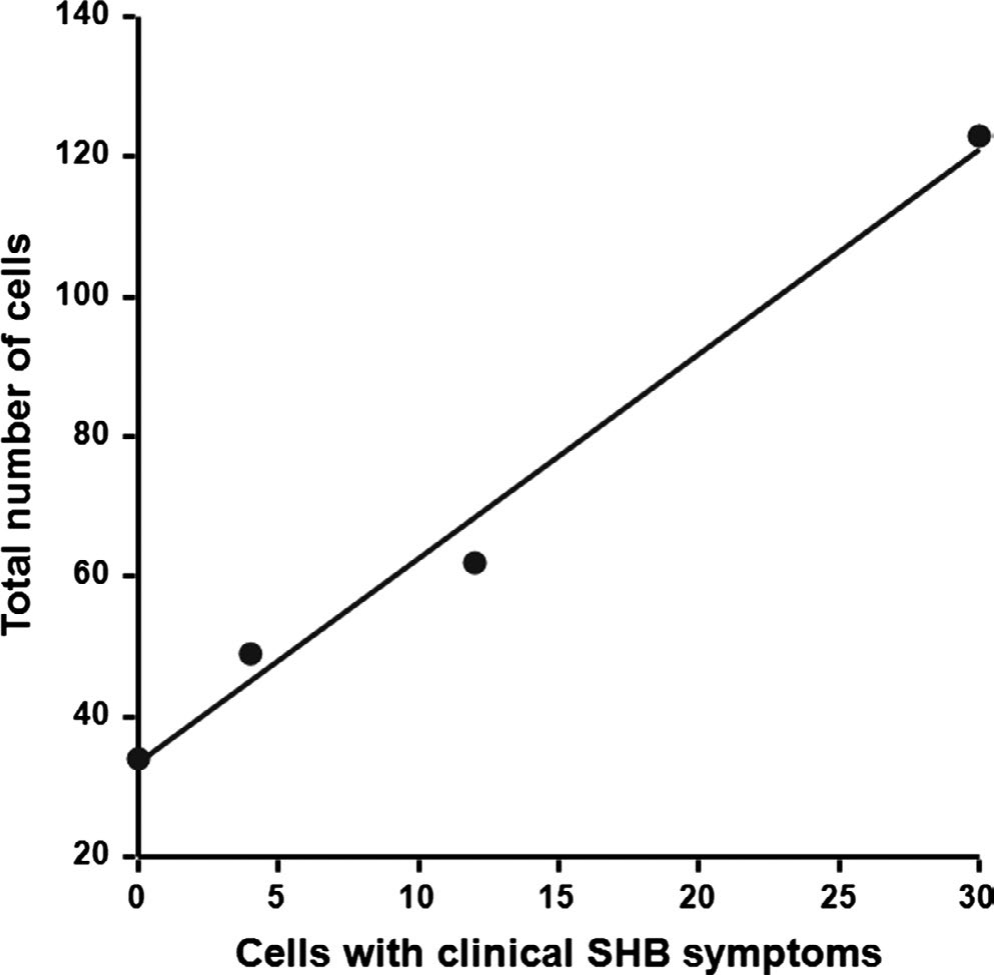
Total number of cells built versus cells with clinical small hive beetle (SHB) symptoms. The correlation is significant (Spearman's correlation, |*r*| (4) = 0.9933, *p* = 0.02). Small hive beetle symptoms positively correlate with the number of cells within a tent. The higher number of cells produced may have amplified the odor cues required for SHB host finding

**Figure 4:**
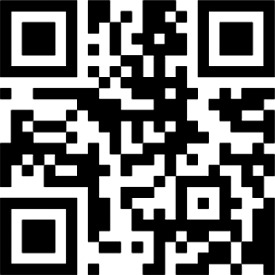
Behaviors of small hive beetles associated with *Megachile rotundata* nests. First part: small hive beetle eating pollen in front of the nest. Second part: small hive beetle larvae crawling around the nest. Third part: small hive beetle larvae eating inside a cell of *Megachile rotundata* during preliminary tests

Three weeks after the establishment of the experiment, one tent from the treatment group was broken due to a storm and was thereafter excluded from the experiment. Upon termination of the experiment, the logistic regression model for the number of nests built by *M. rotundata* did not differ significantly between the control and treatment tents (*χ* ^2^ = 364.32, *df* = 1, *p* = 0.93; Tables 1 and 2). In contrast, the logistic regression model revealed significant differences in the number of nests (*χ* ^2^ = 152.77, *df*= 1, *p* = 0.001; Table 1 and Table 2) and cells (*χ* ^2^ = 36.38, *df* = 1, *p* < 0.002; Table 1 and Table 2) with clinical SHB symptoms between control and treatment tents (Table 1). In some cases (Table 1), SHB eggs were laid outside of cells, resulting in a higher number of nests with SHB symptoms compared to the number of cells. Three of the four treatment tents revealed SHB infestations. Furthermore, our data revealed significant differences in the infestation rates of the treatment tents, whereby tents 2 and 5 showed significantly higher numbers of SHB‐infested nests when compared to tents 3 and 4 (*χ* ^2^ = 38.45, *df* = 3, *p* < 0.001; Table 1 and Table 2). Overall, the percentage of observed nests with clinical SHB symptoms was 41, 12, and 66 for tents 2, 3, and 5, respectively. A significant positive correlation was observed between the number of cells produced per tent and the number of cells with clinical SHB symptoms (Spearman's correlation, |*r*| (4) = 0.9933, *p* = 0.02; Figure 4). This result should however be interpreted with caution, as only four data points were used for Spearman's correlation analysis. In tents 2 and 5, all SHB life stages (Figure 1) were observed; two and five adult SHBs, respectively, emerged from the soil. During the observations, we noticed 11 times between one to two adult SHBs around the nests. Records of two SHB larvae crawling in and out of nests as well as of an adult SHB were made in tent 2 (Figure 3).

**Table 1:** Small hive beetle (SHB), *Aethina tumida,* infestations of *Megachile rotundata* nests in the semifield experiment

Description	Control	Treatment			
	Tent 1	Tent 2	Tent 3	Tent 4	Tent 5
Total number of nests	41	53	41	28	38
Nests with clinical SHB symptoms	0	22	5	0	20
Total number of cells	84	123	49	34	62
Cells with clinical SHB symptoms	0	30	4	0	12
Cells with unspecific clinical symptoms	19	23	9	7	7
SHB adults	0	2	1	0	1
SHB eggs	0	200	0	0	20
SHB larvae	0	6	0	0	1
SHB adults emerged	0	2	0	0	5

Note

The total number of nests (a tube with at least one cell) and cells, nests and cells with clinical SHB symptoms, cells with unspecific clinical symptoms (= not SHB), and the number of observed SHB life stages (parental adults, eggs (shells = 10+ in each case), larvae, emerged adult offspring) are shown for all tents in the treatment (*N* = 50 adult SHBs released) and the control.

**Table 2:** *Megachile rotundata* nests and small hive beetle infestations in treatments and control tents of experiment 2

Variable, *X_i_*	*Z*	*SE* (*Z*)	*p*‐value	Estimated odds ratio	95% CI for odds ratio
Number of nests built between treatment and control group	0.1	0.50	0.93	1.05	0.40–2.70
Number of nests with clinical SHB symptoms between treatment and control group	3.26	35.90	0.001	33.36	4.04–275.05
Number of cells with clinical SHB symptoms between treatment and control group	3.11	28.49	0.002	26.94	3.39–214.09
Number of cells with clinical SHB symptoms between tent 2, 5 and tent 3, 4	−4.93	38.48	<0.001	0.09	0.03–0.23

Note

The results of the logistic regression analysis are shown.

Abbreviations: CI: confidence interval; *SE*: standard error.

Behaviors of SHBs associated with *M. rotundata* nests are shown in a YouTube video (link via Figure 4).

#### 3.1.3 Experiment 3. Will *Aethina tumida* infest and complete its lifecycle in association with *Megachile rotundata* nests under field conditions?

In three of the six locations, ~50% of the cocoons were killed by red fire ants, *Solenopsis invicta*. Therefore, the number of cells built in each location was highly variable ranging from 16 to 214. Upon complete dissection, none of the field nest tubes (total *N* = 157) showed any sign of SHB infestation. However, in three locations, up to five cells were infested by parasitoid wasps of the genus *Melittobia* (2, 2 and 5 cells, respectively).

### 3.2 Association with flowers

#### 3.2.1 Experiment 4. Can flowering plants serve as an alternative food source for *Aethina tumida*?

**Figure 5:**
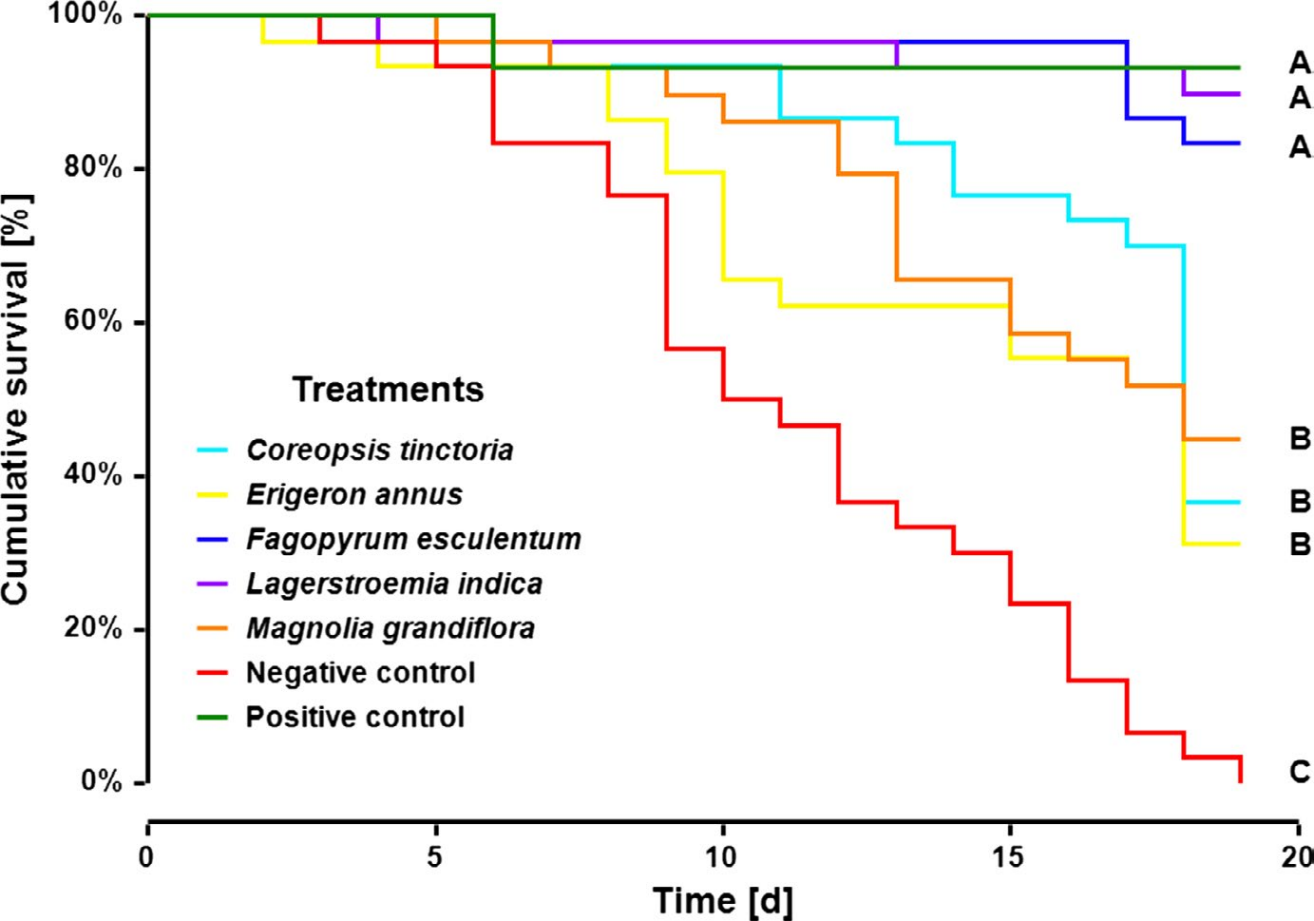
Kaplan–Meier survival curves of adult small hive beetles over the experimental time in association with different flowering plant species, without any food (negative control) and with honey/pollen paste (positive control). Significant differences between groups are indicated by different letters (A, B, C; Logrank test: Mantel–Haenszel, *p* < 0.001)

**Figure 6:**
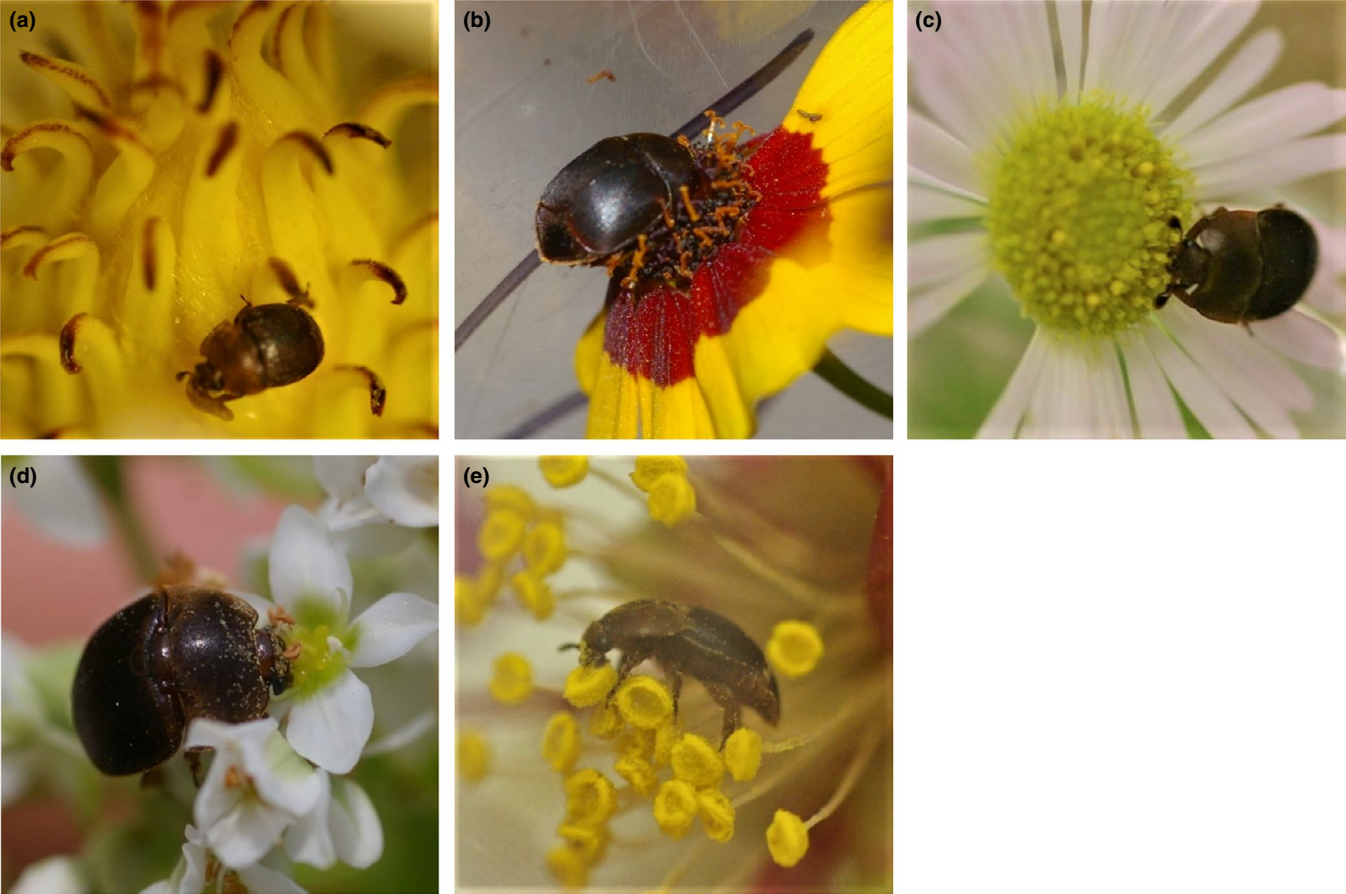
For all plant species, small hive beetle feeding on the flowers was observed at least once over the experiment. Adult foraging on flowers: (a) *Magnolia grandiflora*; (b) *Coreopsis tinctoria*; (c) *Erigeron annus*; (d) *Fagopyrum esculentum*; and (e) *Lagerstroemia indica*

**Figure 7:**
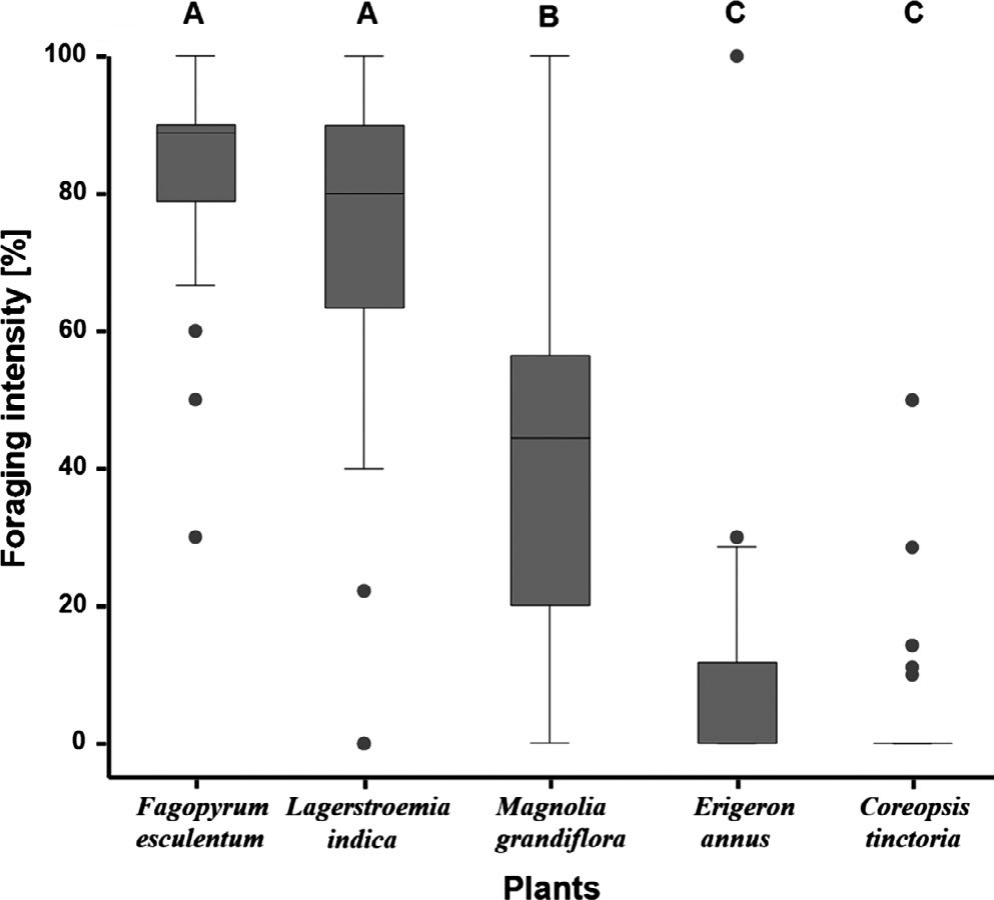
Foraging intensity of adult small hive beetles (SHBs) on each tested plant species measured as the daily proportion of SHBs observed foraging on the flowers (Figure 4) from the total number of SHBs alive in each replicate at this time point. Significant differences among treatment groups are indicated by different letters (A, B, C; Kruskal–Wallis test in One‐Way ANOVA and a Bonferroni test: *p* < 0.05)

**Figure 8:**
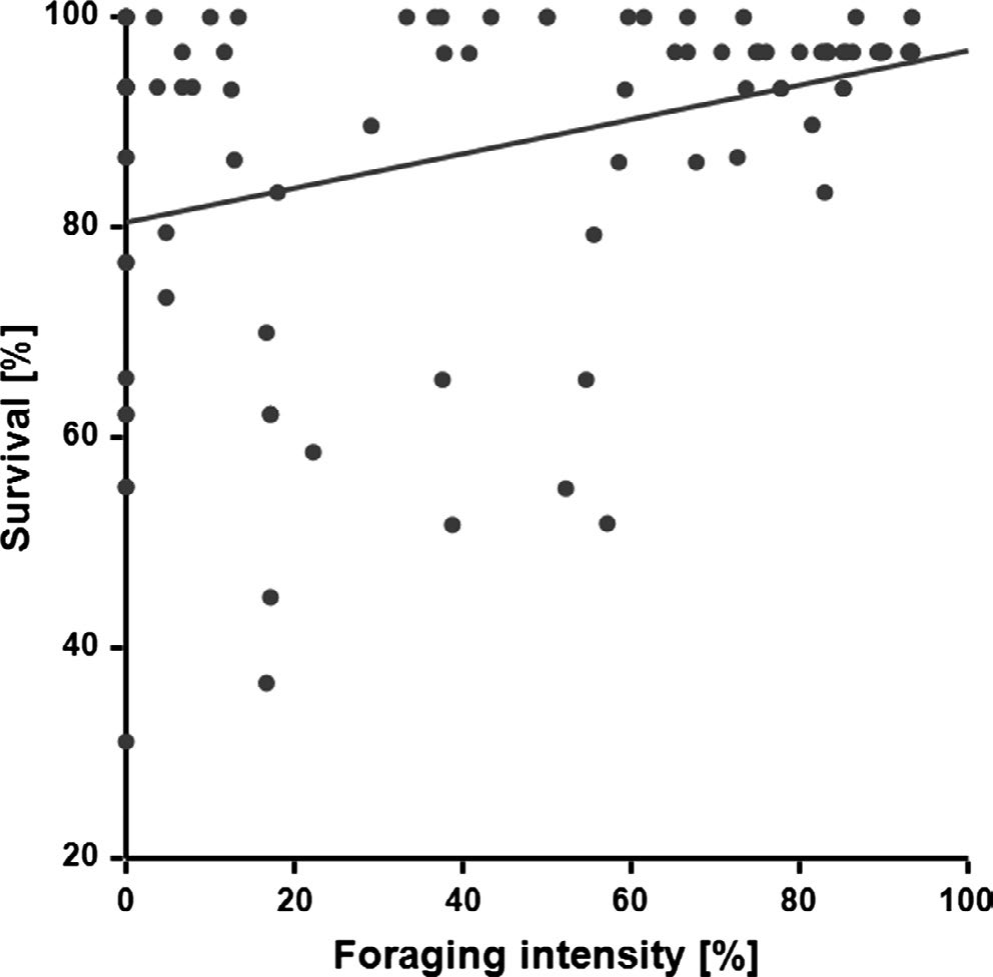
Survival rate of adult small hive beetles versus foraging intensity on the respective flowers. The correlation is significant (Spearman correlation; |*r*| (95) = 0.28, *T* = 2.86, *p* = 0.005)

The negative control revealed the highest mortality and significantly differed from all other groups (Bmtc, *p*‐values < 0.001, Figure 5), with all individuals dying within 19 days. Thereafter, the survival experiment was terminated and the mean survival rates of all groups were compared. No significant differences in survival were observed between the positive control (93 ± 5%) and the plant *F. esculentum* (83 ± 7%) and *L. indica* (90 ± 6%; all *p*‐values > 0.28; mean ± *SD* %; Figure 5). In contrast, the plants *E. annus* (31 ± 9%), *C. tinctoria* (37 ± 9%) and *M. grandiflora* (45 ± 9%) showed a significantly reduced SHB survival when compared to the positive control, *F. esculentum* and *L. indica* (all *p*‐values < 0.001; mean ± *SD* [%]; Figure 5). For all plant species, SHB feeding on the flowers was observed at least once over the 19 days (Figure 6). However, foraging intensity significantly differed between treatments (Bmtc, *p*‐values < 0.005; median [%] ± 95% CI; Figure 7). The foraging intensity ranged between 80 ± 77.8–88.9 and 88.9 ± 80%–90% for *F. esculentum* and *L. indica*, respectively (median ± 95% CI). Their intensities significantly differed from the other three plants *M. grandifloria*, *E. annus,* and *C. tinctoria*, whereby foraging intensity was 44.4 ± 37.5–50 and 0 ± 0 and 0 ± 0 , respectively (all *p*‐values < 0.001; median ± 95% CI; Figure 7). In addition, foraging intensity was significantly correlated with survival rate (Spearman's correlation, |*r*| (95) = 0.28, *p* = 0.005; Figure 8). Lastly, SHB oviposition was only observed in the positive controls.

#### 3.2.2 Experiment 5. Will *Aethina tumida* forage on buckwheat flowers, *Fagopyrum esculentum*, under semifield conditions?

**Figure 9:**
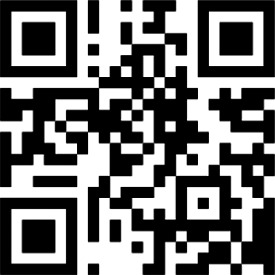
Adult small hive beetle foraging on buckwheat flowers, *Fagopyrum esculentum*

On average, 4.9 ± 0.33 SHB adults were found per infested tent during each observation foraging on the flowers (Figure 9). No SHB was observed at any time in the control tent.

## 4 DISCUSSION

Our study provides clear evidence that SHBs can complete an entire life cycle in association with solitary bees, *M. rotundata*. These solitary bees therefore constitute potential alternative hosts of SHBs, which corresponds with earlier studies suggesting a broad host range (reviewed by Neumann et al., 2016). Field experiments did not show any SHB infestations of *M. rotundata* nests, thereby suggesting that this solitary bee species and possibly other communal nesting ones probably constitute occasional hosts only, for example, when managed or wild social bees are absent. Despite earlier negative evidence (Buchholz et al., 2008; Willcox et al., 2017), our study also clearly shows that adult SHBs forage on flowers under laboratory and semifield conditions, thereby significantly enhancing their survival in the absence of any bee hosts. Taken together, these results support that SHBs are generalists (Torto, Boucias, Arbogast, Tumlinson, & Teal, 2007), which contributes to our understanding of their global invasion success (Neumann et al., 2016) and imposes further practical difficulties for their mitigation (Schäfer et al., 2019).

The preliminary artificial infestation tests with eggs and adults revealed that SHBs are in principle able to infest *M. rotundata* nests due to the presence of clinical symptoms, eggs and larvae. Within the expected periods, the SHB eggs hatched, the larvae reached the wandering stage (~5 days) and adult SHBs emerged from the soil (Neumann et al., 2016), thereby showing that this beetle can complete an entire life cycle in association with solitary bees. The observations showed that adult SHBs laid eggs in *M. rotundata* nests and that larvae fed upon pollen in cells. It therefore appears as if solitary bee nests provide sufficient and adequate food for this parasitic beetle and may constitute alternative hosts, similar to bumblebees and stingless bees (Neumann et al., 2016). However, such manipulative experiments, in which SHBs had no other choice, do not necessarily imply that natural infestations occur. Therefore, both semifield and field experiments were also conducted.

The semifield study further confirmed that SHBs are capable of infesting and completing a full life cycle in association with *M. rotundata* nests. This shows that *M. rotundata* nests are attractive for adult SHBs and that the nests provide sufficient food for successful reproduction. Most likely odor cues of materials common to bee nests (e.g., pollen, Neumann et al., 2016) govern the attractiveness of these solitary bee nests. Overall, the results correspond with previous studies suggesting that non‐*Apis* bee species are alternative hosts of SHBs (bumblebees: *Bombus impatiens*: Spiewok & Neumann, 2006, Hoffmann et al., 2008; stingless bees: *Austroplebeia australis*: Halcroft et al., 2011; *Dactylurina staudingeri*: Mutsaers, 2006; *Melipona beecheii*: Lóriga Peña et al., 2014; *Tetragonula carbonaria*: Greco et al., 2010; Wade, 2012). Interestingly, our data revealed that the infestation rate of SHBs on the *M. rotundata* nests varied significantly among tents. This might be due to varying defensive behavior of the bees, differences in SHB survival rates and/or differences in odor cues. Since clinical SHB symptoms positively correlated with the number of cells within a tent, the higher number of cells produced may have amplified the odor cues required for SHB host finding. However, these results should be interpreted with caution, as only four data points were used.

Defensive behavior toward SHB intruders has previously been described in honeybees, bumblebees, and stingless bees (Elzen et al., 2001; Halcroft et al., 2011; Hoffmann et al., 2008; Lundie, 1940; Neumann et al., 2016), but as not observed in this study, mainly because this was not our focus. However, the discrepancy between the predicted and observed SHB reproduction in the semifield tent experiment (tents 2, 3 and 5), suggests that *M. rotundata* has an efficient defense against SHB infestation. Indeed, many more bee cells showed clinical signs of SHB infestation compared to the SHB life stages actually found during dissections (Table 1). So the number of adult beetles produced underestimates the number of nests attacked.

Our field study revealed that SHBs did not infest any of the experimental *M. rotundata* nests, despite the presence of SHB‐infested honeybee colonies nearby. Due to traps with diatomaceous earth within the colonies (reviewed by Neumann et al., 2016), the overall SHB infestation rate was fairly low, which might have reduced chances for host shifts. Moreover, the rather small solitary bee nests are almost certainly not producing the same amounts of odor cues for attracting adult SHBs compared to honeybee colonies comprised of thousands of individuals and ample food stores. Furthermore, SHBs' distribution within an apiary is not random. Instead, SHBs are known to aggregate in specific colonies suggesting the existence of a respective pheromone (Neumann & Elzen, 2004). Since the colonies were already infested prior to the experiment, short‐range dispersal (Spiewok, Duncan, Spooner‐Hart, Pettis, & Neumann, 2008) may have been governed by the putative SHB aggregation pheromone (Neumann & Elzen, 2004), thereby overriding any kairomones from the *M. rotundata* nests whatsoever. Taken together, it appears as if solitary bees *M. rotundata* may potentially serve as alternative hosts in the absence of honeybees and/or other social bees. Additional comparative field studies are needed to better understand SHB host preference.

Despite earlier negative evidence (Buchholz et al., 2008; Willcox et al., 2017), our observations clearly show that adult SHBs can exploit a wide range of flowering plants, including *F. esculentum*, *L. indica, M. grandifloria, C. tinctoria,* and *E. annuus*, thereby significantly improving their survival. Indeed, survival rates of adult SHBs in association with *F. esculentum* (83%) and *L. indica* (90%) were not different from the positive controls with honey/pollen paste (93%). The significant positive correlation between adult SHB survival and foraging intensity suggests that food availability and attraction varies between the tested plant species. Indeed, pollen and nectar composition and abundance can vary substantially among plant species (Nicolson, 2011; Percyval, 1961; Roulston & Cane, 2000).

Protein content in pollen plays a key role in the pollen consumer's performance (Roulston & Cane, 2000). However, we found no link between the pollen protein content of our plants and the SHB survival rates. For instance, both *M. grandiflora* (23.7%) and *L. indica* (23.1%) have a similar pollen protein content (protein content in [%], Conti et al., 2016), yet revealed significant differences in survival rates, 0.45 and 0.9, respectively. Furthermore, we can exclude that nectar productivity might be a significant factor, as both of the previously mentioned plants do not produce nectar (Thien, 1974). Likewise, we found no link between nectar productivity and SHB survival rates. For instance, *F. esculentum* (0.11 mg/flower) and *C. tinctoria* (0.133 mg/flower; Hicks et al., 2016) produce similar nectar flows per day; however, SHB survival strongly differed, with *F. esculentum* revealing highly better SHB survival rate than *C. tinctoria*, respectively, 0.84 and 0.36. Therefore, other factors may have played a role, such as the production of toxic molecules (Adler, 2000), the mechanism and efficiency of digestion (Roulston & Cane, 2000), as well as the pollen and nectar accessibility (van Rijn & Wäckers, 2016) and attractiveness of plant volatiles (Pichersky & Gershenzon, 2002). Floral volatiles serve as attractants for pollinators (Pichersky & Gershenzon, 2002) and may have increased the likelihood of the SHBs to visit the flowers. Decanal, for instance, is a characteristic component of buckwheat aroma (Janeš, Kantar, Kreft, & Prosen, 2009) and interestingly, this molecule is also produced by honeybee workers (Torto et al., 2007) and may attract adult SHBs (Stuhl & Teal, 2017). The disparity with the results of the study of Buchholz et al. (2008) could be due to the different plant species used within either study. Other reasons might be the differences in experimental designs and/or tested beetles. While Buchholz et al. (2008) used freshly emerged adults, the tested adult SHBs in our design were fed with honey/water for at least 10 days prior to the experiment (see above). Indeed, SHB adult mortality in both positive and negative controls was higher in the Buchholz et al. (2008) study. Since there are >12 years between the conducted studies as well as >1,000 km between the tested SHB populations (Beltsville, MD, USA Buchholz et al., 2008, Auburn, AL, USA), spatio‐temporal differences in insect populations may well contribute (e.g., as in *Stilbosis quadricustatella* leafminers, Mopper, Stiling, Landau, Simberloff, & Zandt, 2000).

Similar to an earlier study (Buchholz et al., 2008), no SHB oviposition was observed in association with any of the tested flowers despite amply eggs being laid in the positive controls (> 20 per replicates). This suggests that SHB females do not regard flowers as a suitable breeding substrate in contrast to meat and fruits (Buchholz et al., 2008; Ellis et al., 2002). Alternatively, but not mutually exclusive, the pollen obtained from the flowers may have not been adequate for full activation of the female ovaries (de Guzman, Rinderer, & Frake, 2015). Obviously, female SHBs cannot produce an infinite number of eggs. It therefore appears adaptive to limit reproduction to optimal breeding grounds (Neumann et al., 2016). Nonetheless, the results of the laboratory experiment suggest that flowering plants are a sufficient nutritional resource for adult SHBs to survive at least 19 days and probably longer when bee hosts are not available. This was confirmed by the semifield experiment, where adult SHBs were frequently observed foraging on buckwheat flowers in the tents despite the close proximity of solitary bee nests. This is consistent with former findings that SHBs seem to be opportunistic and are in principle able to exploit a wide range of alternative food sources (Buchholz et al., 2008; Ellis et al., 2002). The observed association of SHBs with flowering plants is not surprising because very closely related species, for example, *Aethina concolor* are known to be flower visitors (Buchholz et al., 2008).

## 5 CONCLUSIONS

In general, our study provides strong support for the opportunism of SHBs, thereby contributing to our understanding of the global invasion success of this species. Flowers are very likely to serve as alternative food sources for adult SHBs in the absence of bee hosts. Even though flowers do not appear to elicit SHB reproduction, the provided food may nevertheless foster SHB invasion success. Furthermore, SHBs were capable of completing an entire life cycle in association with solitary bee *M. rotundata* nests. Even though SHB infestations of solitary bee nests were not observed in the field, our observations nevertheless create demand for field studies on the role of wild solitary and social bee species for the success and possible impact of this biological invasion. Finally, the possible role of flowers as alternative food and of solitary bees as alternative hosts imposes further practical difficulties for adequate mitigation of this invasive species.

## CONFLICT OF INTEREST

None declared.

## AUTHOR CONTRIBUTIONS

J.G., L.S., and P.N., wrote the manuscript and designed the experiments; G.R.W. and P.N. provided laboratory and material; J.G., A.P., and P.N. collected field and laboratory data; L.S. conducted the statistical analysis. J.G., L.S., and P.N. analyzed the data. J.C. helped setting‐up the experiments with flowers. All Authors edited and approved the manuscript.

## Data Availability

The complete raw data can be found at the Dryad repository. See (DOI: https://doi.org/10.5061/dryad.cg5p7bk.)
